# Technical evaluation of a clinical, bi‐planar, digital and upright X‐ray imaging unit

**DOI:** 10.1002/jmrs.519

**Published:** 2021-06-06

**Authors:** Charlotte Kelly, Ioannis Delakis

**Affiliations:** ^1^ Radiology Department Sidra Medical and Research Center Doha Qatar; ^2^ Radiology Department Weill Cornell Medical College NY USA

**Keywords:** data collection, phantoms, imaging, quality control, radiation protection, technology, radiologic

## Abstract

We describe the technical evaluation of the commercially available, clinical, bi‐planar, low dose, digital X‐ray system (EOS System, EOS imaging, France). The unit is used for upright, weight‐bearing musculoskeletal pathologies, in particular, in the spine and lower limbs. The evaluation incorporated tests on the X‐ray generator performance, radiation/imaging field alignment, dose area product accuracy and image quality. The assessment methodology was based on objective parameters and required equipment readily available for technical evaluation of other radiological equipment. Results demonstrated that the system performs well within acceptable performance criteria with regard to X‐ray generator performance, radiation/imaging field alignment and dose area product accuracy. In addition, results from the image‐quality assessment were aligned with previously published work. The work presented in this article can be used for the technical evaluation of the EOS System at other clinical sites.

## Graphical abstract

Our article describes the technical evaluation of the EOS System, which is a new radiological imaging equipment used for bi‐planar, upright radiological studies, adopting novel detector design. In our work, we adapted conventional performance assessment methods to evaluate the system's X‐ray generator and dose indicator. We also incorporated the measurement of generalized values of quantitative image quality metrics, in order to recreate a more clinically relevant assessment process. The methodology and results of our work can be used by other clinical sites with the same equipment for the purposes of acceptance, commissioning and quality control testing of the equipment.

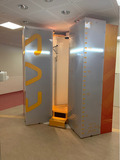

## Introduction

Technical evaluation of radiological equipment installed in clinical sites is an integral part of the radiology quality assurance framework and a legislative requirement, ensuring the safe and optimised use of radiation for clinical imaging. Professional bodies have published guidelines on technical evaluation and commissioning methodologies for radiological equipment, for example, the European Commission has summarised a number of these tests and the proposed performance acceptability criteria in an extensive report.^1^ However, these recommendations and guidelines cannot be applied in a straightforward manner for specialised radiological units such as the EOS System (EOS imaging, France), on account of its unique design.

The EOS System has found broad use in hospitals and orthopaedic specialty clinical centres for spinal and lower limb examinations. Previous work has shown that the EOS System has a strong potential for dose saving in patient studies compared to conventional digital radiography imaging, which can be even further reduced in its microdose function.^2^, ^3^ The unique technical characteristics and functionality of the system allow for significant dose reduction, which is an important consideration in particular for paediatric orthopaedic examinations which may require frequent follow‐ups.^4^, ^5^, ^6^

The purpose of the work presented in this article is to demonstrate the methodology adopted by our team to perform the technical evaluation and commissioning for clinical use of the EOS System, and to present baseline values that can be applied as performance acceptability criteria.

## Material and Methods

### EOS system

**Figure 1:**
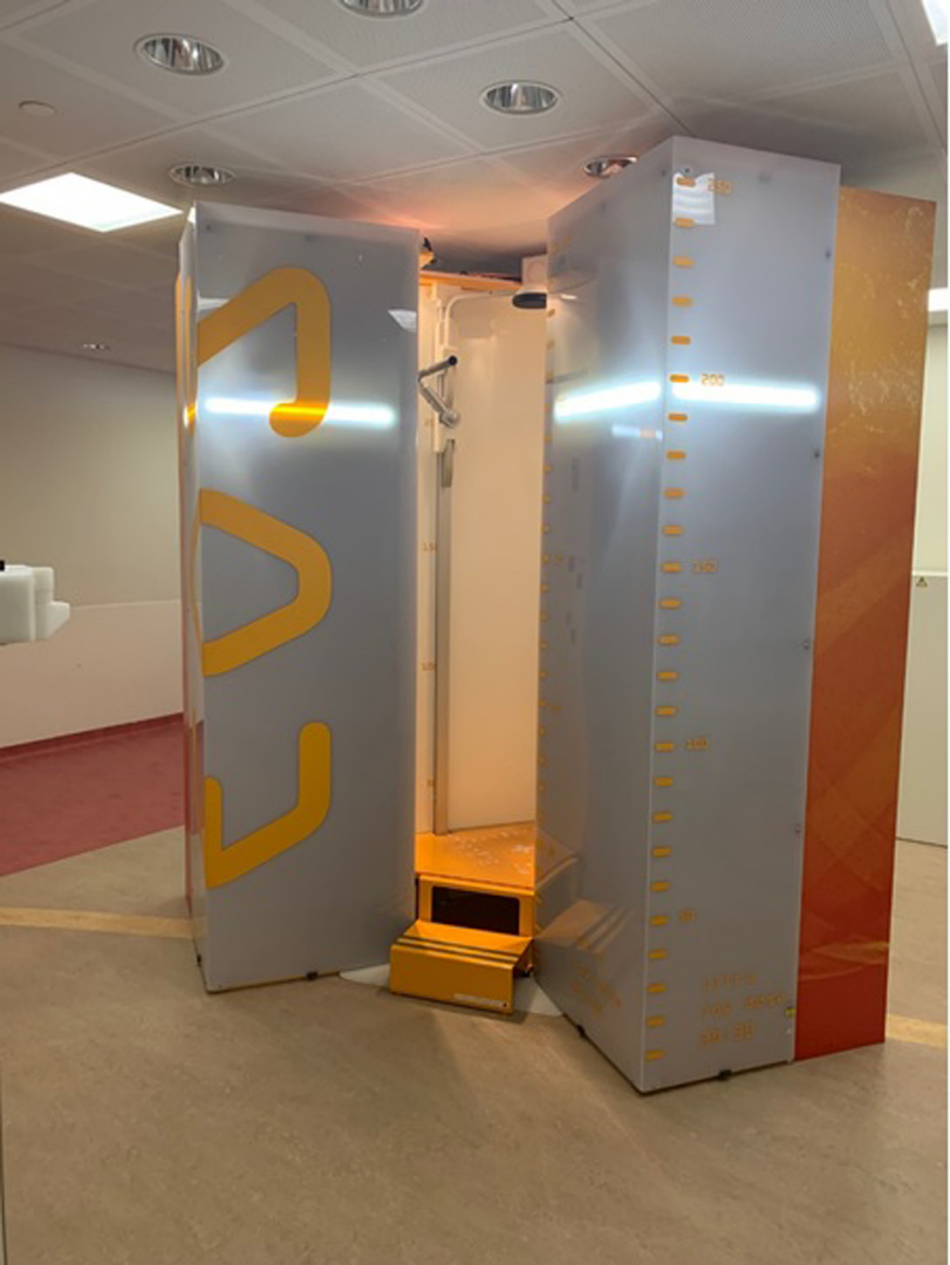
EOS System (EOS imaging, France) installed in our hospital. Patients step onto the central platform of the system where they stand whilst the image is acquired.

**Figure 2:**
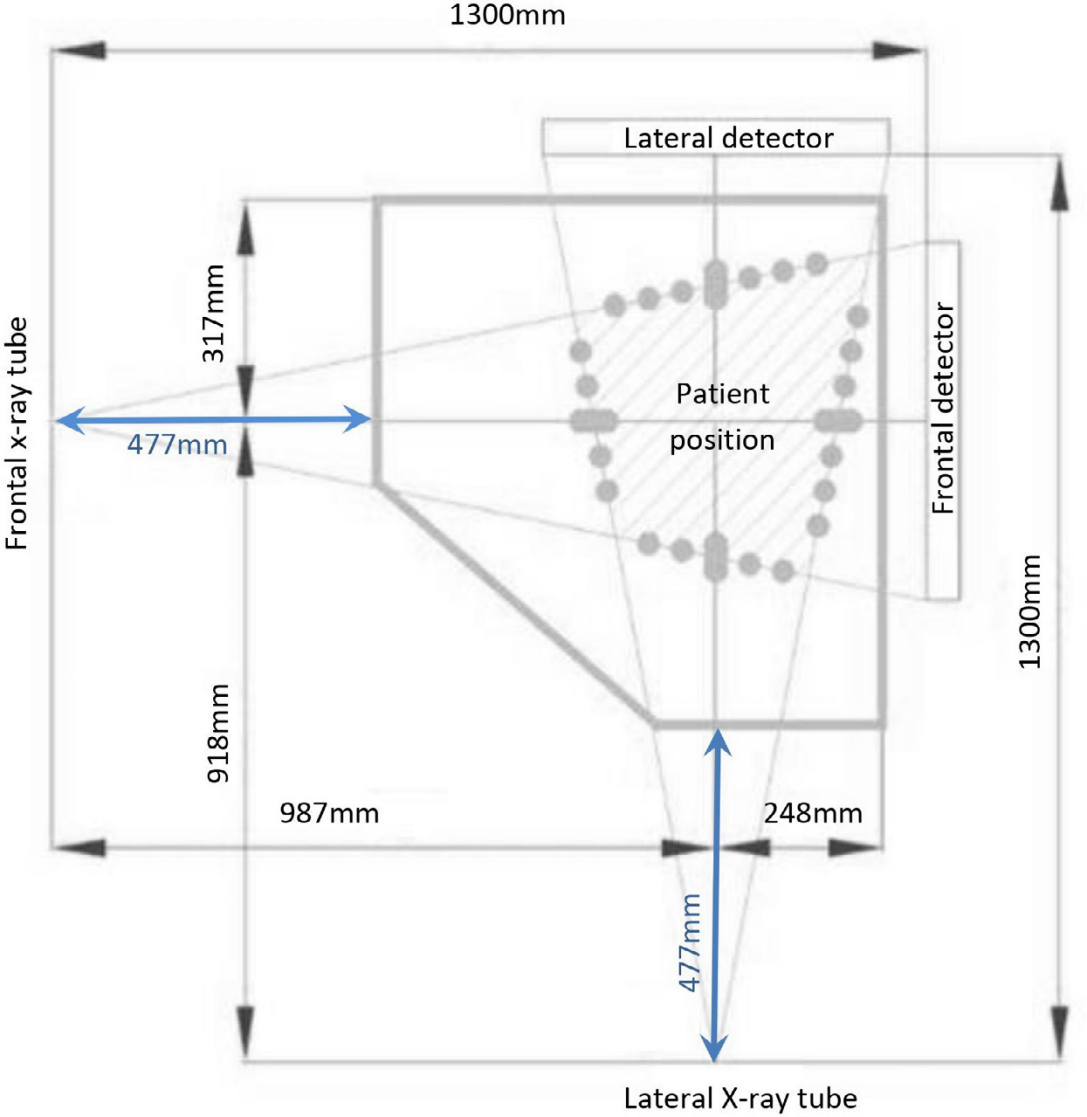
Geometry of the EOS System (view from above) indicating the Source to Image Distances (SID) for each X‐ray tube/detector combination (frontal and lateral). The patient stands in the middle of the central platform of the system. The distance between each X‐ray tube and the EOS system covers nearest to the tube is indicated in blue.

The EOS System is shown in Figure 1 and its geometry depicted in Figure 2. Each X‐ray tube/detector combination moves vertically at different lengths, as adjusted for each study by the operator, to obtain frontal/anteroposterior (AP) and lateral (LAT) images simultaneously. The distance by which the tube travels vertically during image acquisition for each study will henceforth be referred to as study length.

The X‐ray sources of the unit consist of two fan‐shaped X‐ray beams collimated to 0.5 mm width. The beams are positioned at 90° to each other and coupled to linear detectors with 0.5 mm‐thick aluminium collimators, to prevent scattered radiation from detection.

The detector design is based on a gaseous X‐ray detector with a proportional multi‐wire chamber.^7^

### X‐ray generator performance assessment

**Figure 3:**
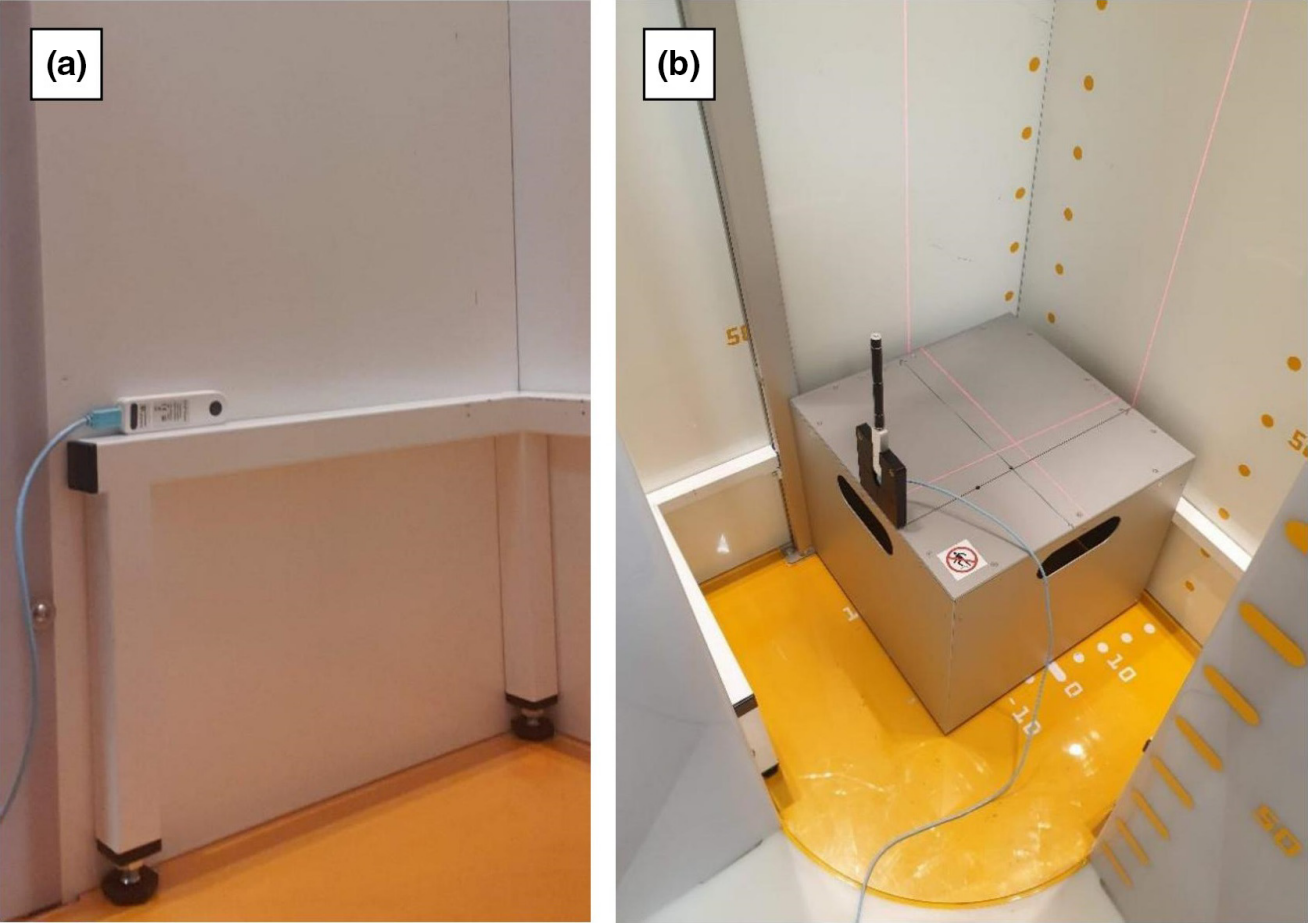
(A) Dosemeter (R/F chamber) position for generator performance tests of the frontal X‐ray tube. (B): Dosemeter (pencil chamber) position for dose area product (DAP) indicator accuracy assessment.

The X‐ray generator was evaluated using a calibrated RaySafe X2 dosemeter (RaySafe, Sweden). The ‘R/F detector’ of the dosemeter was secured against the imaging system’s covers closest to the frontal X‐ray tube and aligned with the centre of the X‐ray tube using the system’s positioning lasers (Fig. 3), resulting in a tube‐to‐dosemeter distance of 47.7 cm for both X‐ray tubes, as also indicated in Fig. 2. Exposures were made at different kV values (60 kV to 100 kV at increments of 10 kV), with a set mA value of 200 and filtration (0.1 mm of Cu), and the kV, radiation output and half‐value layer (HVL) values were recorded using the RaySafe X2 dosemeter for each measurement. The ripple of the kV waveform was used in estimating inferred filtration.^8^ The process was repeated for the lateral X‐ray tube. Results from the measurements were compared against remedial (error ±5 kV, accuracy 5%) and suspension levels (error ±10 kV, accuracy 10%) based on professional guidelines for X‐ray tubes used in medical radiography.^9^

### Radiation/image field alignment assessment

The radiation field of the EOS System was captured by placing gafchromic film (Ashland, USA) sensitive to general radiology dose range (0.1–20 cGy) within the imaging field, on the covers of the EOS System closest to the detector. Both dimensions of the radiation field were compared against the respective values indicated by the system for the purposes of ensuring that radiation and image field sizes coincide, using a tolerance of ±1 cm (at reference source‐to‐image distance of 1 m).^9^

### Dose area product accuracy assessment

In order to measure the DAP, the 10 cm pencil ionisation detector of the RaySafe X2 dosemeter was placed at a distance of 27.5 cm from the imaging system’s covers closest to the frontal X‐ray tube and aligned with the centre of the X‐ray tube using the system’s positioning lasers (Fig. 3). The use of the pencil ionisation chamber allows for continuous recording of the radiation dose whilst the X‐ray tube is moving during the clinical mode of operation. In addition, placing the pencil ionisation chamber closer to the X‐ray tube allows for more accurate recording of the system’s low radiation output and makes the measurement less sensitive to sources of scattering from the imaging system.

Ten exposures were made for each patient morphotype default exposure settings available on the system (thin, average and large patient), as shown in Table 1. Changing the patient morphotype allows for the assessment of DAP accuracy across different X‐ray tube outputs. Patient morphotype can be selected by the operator depending on the size of the patient presenting. The study length was adjusted to 5 cm to ensure that the radiation beam is captured in its entirety by the pencil ionisation detector. The width was also kept at 5 cm to minimise potential variability of dose along the width of the radiation area. The dose length integral was measured along with the pencil chamber as the X‐ray beam was moving from −2.5 cm to +2.5 cm, taking as zero the middle point of the chamber. The dose length integral measured was multiplied by the width of the radiation area in correspondence with the pencil chamber to provide the measured DAP value. The process was repeated for the lateral tube. A tolerance value of 25% was used for deviation between measured and indicated DAP values.^10^

**Table 1:** Dose area product (DAP) results.

Patient morphotype	kV	mAs	Speed setting	Indicated DAP (mGycm^2^)	Measured DAP (mGycm^2^)
Frontal tube					
Thin	83	200	4	1.48	1.6 ± 0.2
Normal	90	250	4	2.20	2.3 ± 0.3
Large	100	250	4	2.74	2.8 ± 0.3
Lateral tube					
Thin	102	200	4	2.31	2.5 ± 0.3
Normal	105	250	4	3.07	3.3 ± 0.3
Large	110	320	4	6.02	5.9 ± 0.4

### Image‐quality assessment

Image‐quality assessment was based on estimating the generalised detective quantum efficiency (GDQE), which describes spatial resolution and noise properties of an imaging system under clinically appropriate conditions. ^11^, ^12^, ^13^ By using the Generalised Modulation Transfer Function (GMTF) and Generalised Normalised Noise Power Spectrum (GNNPS) we can calculate the Generalised Noise Equivalent Quanta (GNEQ) as:

(1) $$\mathrm{GNEQ}\left(f_{x}{,f}_{y\;}\right)=\frac{{\mathrm{GMTF}(f_{x}{,f}_{y\;})}^{2}}{\mathrm{GNNPS}(f_{x}{,f}_{y\;})}$$

where *f_x_* and *f_y_* is spatial frequency in *x* and *y* directions respectively. The GDQE can then be calculated as:

(2) $$\mathrm{GDQE}\left(f_{x}{,f}_{y\;}\right)=\frac{\mathrm{GNEQ}(f_{x}{,f}_{y\;})}{q}$$

where q is the ideal squared signal‐to‐noise ratio per unit exposure that can be provided by the system given the number of incident X‐ray quanta.

The phantoms used for GNNPS and GMTF analysis were two clear, square, polymethyl methacrylate (PMMA) plates of 5 cm thickness and a copper edge of 0.5 mm thickness. GNNPS was measured from four images acquired with the two PMMA plates positioned against the covers of the system on the detectors’ side. GMTF was measured on an image acquired by placing the copper edge between the two PMMA plates, at a slightly oblique angle (1‐2°) and imaging at the same location. GNNPS and GMTF include the effects of detector blur, focal spot unsharpness, magnification and scatter properties of the system.

The value of q in equation (2) was estimated by calculating the dose to the detector from measuring the dose attenuated by the PMMA and distance‐corrected to the position of the detector. The number of incident X‐ray quanta was then calculated based on the work by Boone and Seibert.^14^

## Results and Discussion

As explained in the description of the EOS system, the X‐ray tube/detector combinations move vertically in order to acquire clinical images. The methodology described in this article proposes keeping the X‐ray tube stationary only for the purposes of testing the X‐ray tube and generator performance and, in that respect, it is aligned with existing evaluation guidelines.^9^ However, DAP accuracy evaluation and quantitative image‐quality assessment are applied in clinical mode, whereby the unique function of the system and detector design limits the application of methodologies described in existing evaluation guidelines. ^10^, ^15^ In this context, the methodology proposed in this article can provide users with an alternative, applicable test protocol to evaluate the system. As the methodology has been developed to measure the same performance parameters as in existing evaluation guidelines, it is proposed that the same tolerance criteria are adopted.

### X‐ray generator performance

The results from the X‐ray generator performance assessment are shown in Table 2.

**Table 2:** X‐ray generator performance results

Parameter	Frontal tube	Lateral tube	Remedial level	Suspension level
kV error (kV)	−3.4	0.9	±5	±10
kV accuracy (%)	0.03	0.01	±5	±10
Output repeatability (%)	0.02	0.05	Mean ±10%	Mean ±20%
Specific radiation output (µGy/mAs)	29.4	29.2	Baseline ±20%	Baseline ±50%
Inferred filtration (mmAl) at 80 kV	6.4	6.7		
Half‐Value Layer (mm Al) at 80 kV	5.11	5.16		

The kV error, kV accuracy and output repeatability results were all within acceptable ranges. The HVL and inferred filtration values incorporate the 0.1 mm Cu additional filtration which cannot be removed when imaging. The additional filtration of the EOS System was also identified in previous work and it contributes to the low‐dose level of the system whilst still allowing for adequate contrast at skeletal imaging where high intrinsic contrast is present (bone versus soft‐tissue).^16^ The specific radiation output results for the EOS System fall within but near the lower end of the 26–43 µGy/mAs range indicated in IPEM Report No.32 Part 1.^17^

### Radiation/image field alignment assessment

The alignment of the radiation field with the laser positioning lights and the exposure planning indicators was well within the 1 cm tolerance on all four dimensions of the field,^9^ indicating that a lower tolerance of less than 0.5 cm could be used instead by the user as a more stringent remedial level for the technical evaluation of the system.

### Dose area product accuracy assessment

Results from comparing the indicated and measured DAP values for the frontal and lateral tube are shown in Table 1 and the deviation between values was in all cases well within the 25% tolerance level.^10^

### Image quality

**Figure 4:**
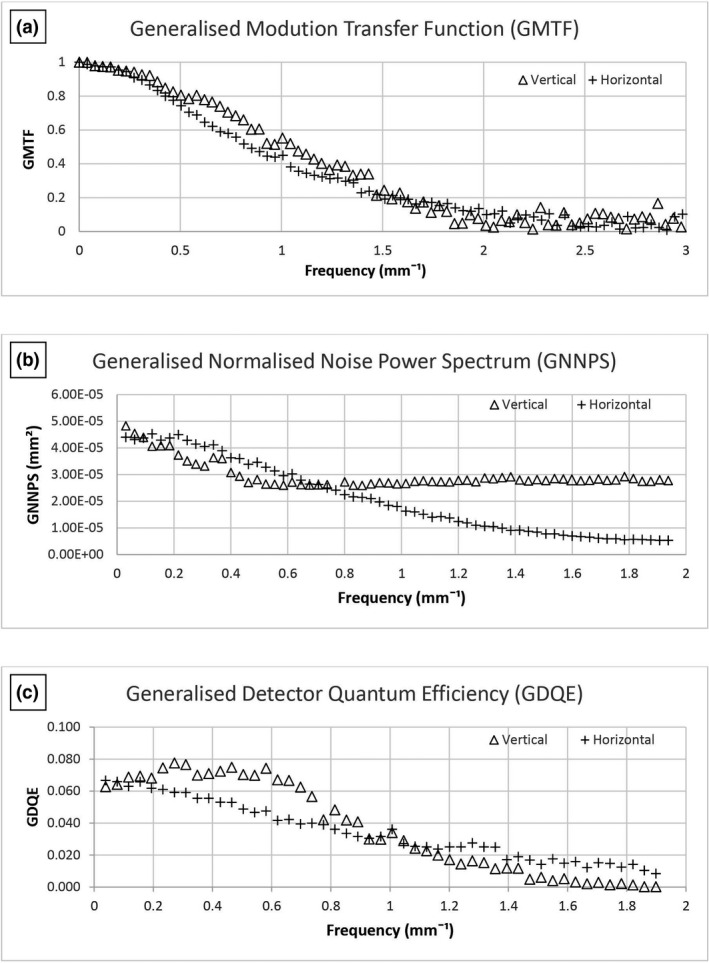
(A) GMTF, (B) GNNPS and (C) GDQE for the frontal X‐ray tube and detector pair.

**Figure 5:**
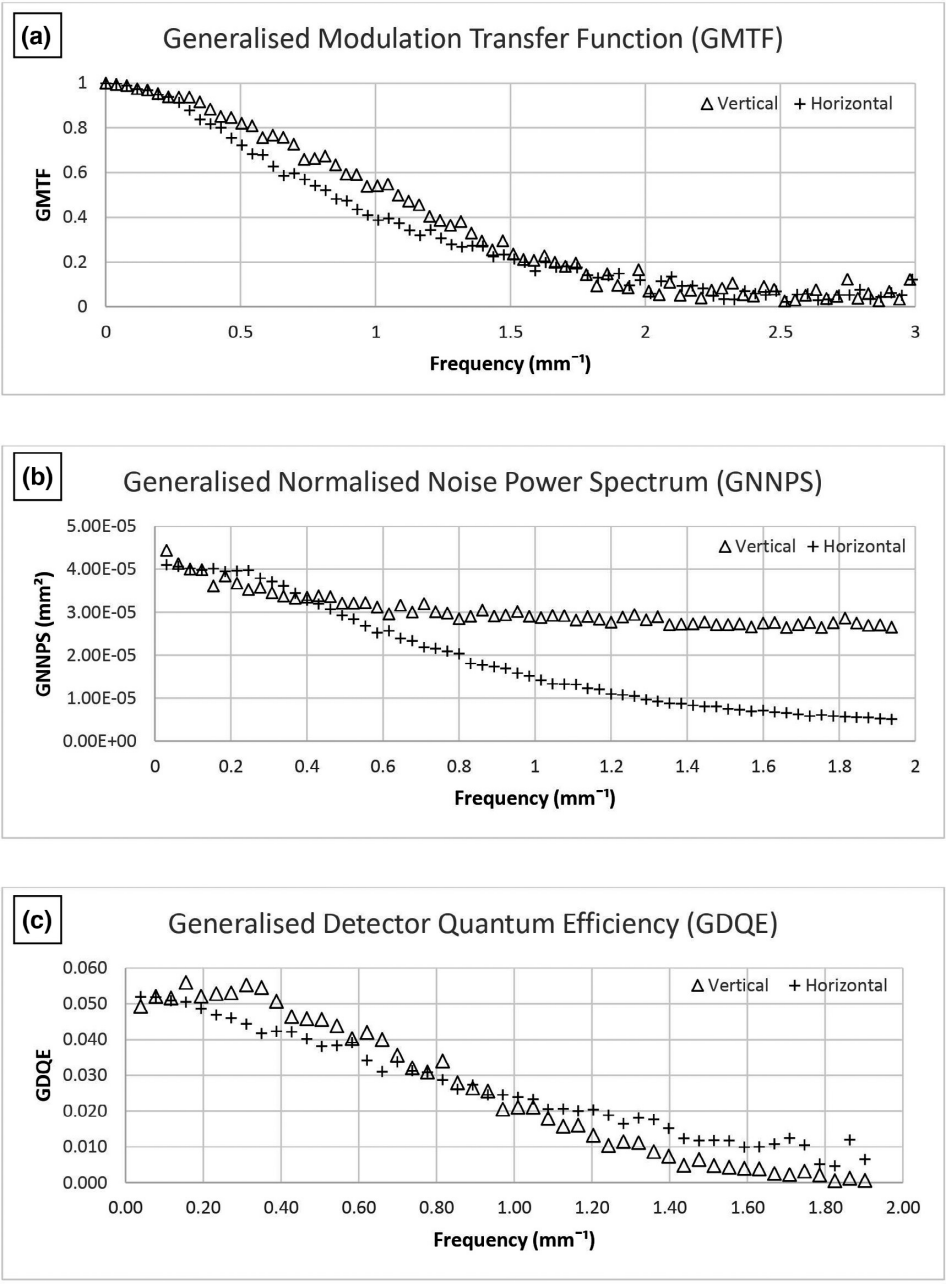
(A) GMTF, (B) GNNPS and (C) GDQE for the lateral X‐ray tube and detector pair.

GMTF, GNNPS and GDQE results are shown graphically in Figures 4 and 5 for the frontal and lateral tube/detector combinations, respectively. The vertical direction is the one that the X‐ray tube moves during acquisition (scan direction) whereas horizontal is the direction of the detector slit (slit direction). The GMTF in the horizontal direction is influenced by signal scattering between pixels, as photoelectrons are able to travel in the horizontal direction and contribute to the signal in neighbouring channels. On the other hand, the line‐by‐line acquisition in the vertical direction prevents photoelectrons contributing to neighbouring channels in this plane.^16^

GNNPS results show that they are independent of the spatial frequency in the vertical direction as there is no pixel correlation, whereas in the horizontal direction, the charge sharing process between adjacent channels produced pixels correlation and noise is expected to decrease as a function of frequency.^16^

The eDQE differences between the vertical and horizontal directions are attributed to the image acquisition process, as discussed in relation to the GMTF and GNNPS results.

## Conclusions

Technical evaluation of the EOS System can be performed by following the proposed methodology and the data presented can be used for comparative purposes or baselining. The system performed well within acceptable performance criteria. There were differences in image‐quality specific results, depending on the imaging direction studied, but these results were anticipated from the system design. Results also showed that it was appropriate to adopt performance tolerance criteria of existing evaluation guidelines, even though an alternative test protocol was implemented. The proposed methodology requires set‐up time on the system and off‐line data processing which may not always be readily available. Therefore, it is recommended that subjective image‐quality tests tailored to the needs of each clinical site are performed as an adjunct to the proposed methodology, in order to establish local baselines for quick, periodic quality control of the system.
